# Supplementary material for: Functional Characterization of the MeSSIII-1 Gene and Its Promoter from Cassava
Source: Int J Mol Sci. 2024 Apr 26;25(9):4711. doi: 10.3390/ijms25094711 (PMC11083483; doi:10.3390/ijms25094711)
Supplement: Supplementary file 1 [file ijms-25-04711-s001.zip › ijms-2953032-supplementary.pdf]

Supplementary Date

Supplementary Figures

-1228 ATTTTTAAATCTTTTATTATGGATTTAGATTTTTAAAATTAGTTATTTC
ERF

-1178 AATAGATTTTTTATATTTAGACCTTAGAAATCTCTCTATTTTTCAGATAA

-1128 GTTTTGAGCCTTTTAACATATAAGTTTTTAAATATTATGTTAATTTTGGG

-1078 AATATTTTTTATTCATCTAATGAATATTATTATCTTAAATAAAAAAAAT

-1028 TGTTGACATGAAATAATTATTGCACTATATATTTTTTCCATTTTCCTGCG
O^{2-}site

-978 AATTTGTAACAAAGGAAAAGGAAAAAAGATCCTACTAATGAGCTAATATT
GATA-motif

-928 TAGTACCATGTAGTGAAAAGATTTAAAATTATAATATAATAATAACATAA
ERF

-878 TGGCATTTAAATTATATGAAAATTGTAAAATTATAATGTATGATTTTAAA
ERF

-828 AAAGAATTCAAATAACTTTCATATATGATTAAAACAATTTTTAAATTTAA

-778 AAAAATAAATTAATTACTGTAATCATTCATTGGCATTGTCATCATCATCA
Box 4

-728 TTATTATTGCTATAAATAAATATTATAATAAAAACTATTATCCCTTAACA

-678 TCACTTAACATTTCTTTTTACAACTCACTTCATTTAGAATGAGTTATTTT

-628 GAAAAGAAATAATTTTTATAAAATTTTATTTTTTTTAAAATAAAATAAAA
ERF

-578 TATTCTTTTATTTCAACTAATAAAAAATAATTAAATTAAATTAAATTAAA
AT1-motif

-528 TATCAGCCAATGTTTCAAATTGGAGTTAAATATATGAAAAATCGATGGTTT
ARE

-478 TTCCCCACAAGAGTTGGTGAAAAGATTTACTTTACCTATTATTTTATAG

-428 TAGACTCCAACAGCAAGAGCGCCAACGTGGCAAATATTTATGACGTATCA
G-Box/ABRE TGACG-motif

-378 CTTCCAACGAATCTAAGCCTATCATCAGATCACAGATGGATAATAGCCTT
ATCT-motif

-328 AAAAACATTAAAATACCGAATTATTTATTCTTAAAAAAAATTTTAAAATT
ERF

-278 ACAAAAAAAAAAATTACATGCCGCTTATCTTTGTACAAGATCTTATCTTTG
GATA-motif

-228 TACAAGATCACACAATGGACGGCACAAAAAGCTACCGCAATGTTCACTGG

-178 AACGTAGCAATCAACGGTCAGTATGCTGCGCATTTGTTAACATTGTAGGC
CCAAT-box

-128 TGCATCGTAGAAGATTACAATCATTATTATTAATAGTTATGGCTGATATT
Box 4

-78 CTTGCTACTCCAGTGCATTCTTGTTGTTAGTGAGAAACTGAGAACAATTT

-28 TCGATTTCTGAGTTGGTAACCGTCAGCCATGGAAGTGGGTTTGCAGGT
CGTCA-motif

Figure S1. Physical map of *MeSSIII-1* promoter. The "A" of translation initiation code "ATG" of *MeSSIII-1* was designated as "+1". The TATA-box is highlighted in bold. Putative *cis*-acting elements are underlined, colored and labeled. The *cis*-acting elements sites on the positive strand and the negative strand are shown in red and blue, respectively.

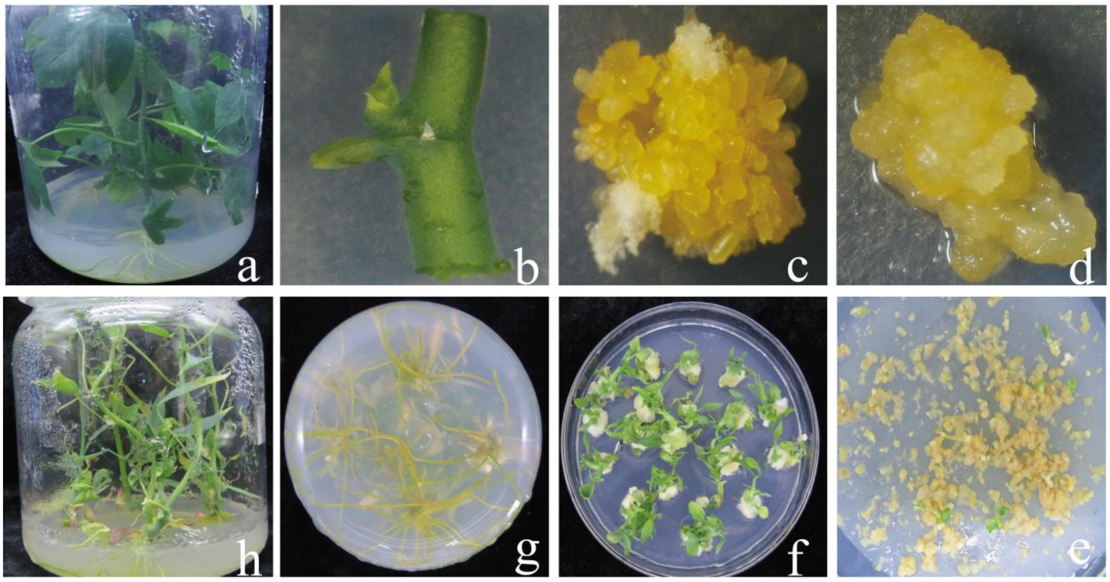

Figure S2. *Agrobacterium*-mediated genetic transformation of cassava SC8 FECs
a Aseptic culture seedling. b Axillary bud. c Somatic embryo. d Fragile embryonic calli. e Induction of resistant cotyledons. f Induction of regeneration shoots. g Rooting Screening. h Regeneration of resistant plants.

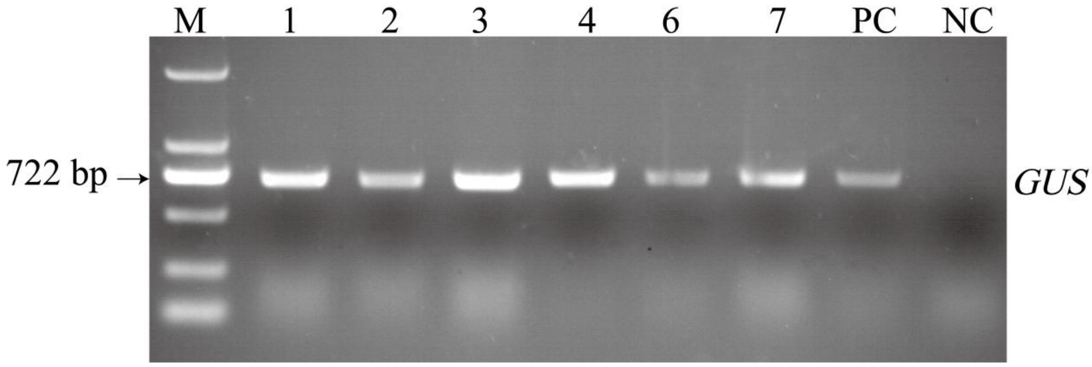

Figure S3. PCR identification of the proMeSSIII-1 transgenic plants by *GUS*.
M: 2000 DNA marker. 1-7: transgenic plants. PC: positive control. NC: negative control.

Supplementary Tables

Table S1 Primers used in this study

**Table S1** Primers used in this study		
**Primer name**	**Primer sequencess (5′→3′)**	**Purpose**
qMeSSIII-1-F	TGGGTTTGCAGGTACACAGA	For qPCR
qMeSSIII-1-R	GGAAAGACCCAGAGAGACGA	For qPCR
qTUB-F	ATGCGGTTCTTGATGTTGTTC	For qPCR
qTUB-R	TCGGTGAAGGGAATACAGAGA	For qPCR
MeSSIII-1-F	TGAGTTGGTAACCGTCAGCC	For *MeSSIII-1*gene amplification
MeSSIII-1-R	ACAAATATGGTGTGCGGGGT	For *MeSSIII-1*gene amplification
1300-MeSSIII-1-F	tgatacatatgcccgtcgacATGGAAGTGGGTTTGCAGGTAC	For construction
1300-MeSSIII-1-R	tgggtaggatccggtaccTGAGAACCATGACCTGTGGG	For construction
1300-F	CGGGGGACTCTAGATACATCA	For vector detection
1300-R	CTTGTGGCCGTTTACGTCG	For vector detection
proMeSSIII-1-F	CTCTTCTATTCTGCCTGA	For promoter amplification
proMeSSIII-1-R	TGAGGGAAAGACCCAGAG	For promoter amplification
SP0-F	GGATCCTCTAGAGTCGACATTTTTTAAATCTTTTATTATGGA	For construction
SP1-F	GGATCCTCTAGAGTCGACTTTCCTGCGAATTTGTAACAAAGG	For construction
SP2-F	GGATCCTCTAGAGTCGACGGCATTGTCATCATCATCATTAT	For construction
SP3-F	GGATCCTCTAGAGTCGACATCGATGGTTTTTCCCCACA	For construction
SP4-F	GGATCCTCTAGAGTCGACACATGCCGCTTATCTTTGTACA	For construction
SP-R	CTTTACTAGTCAGATCTACCATGGCTGACGGTTACCAACT	For construction
GUS-F	CCTCGCATTACCCTTACGCT	For identification
GUS-R	TTTCTTGTTACCGCCAACGC	For identification

Table S2 Main *cis*-acting elements in *MeSSIII-1* promoter

***Cis*-elements**	**Number**	**Function**	**Core Sequence**
CAAT-box	22	common cis-acting element in promoter and enhancer regions	CAAT
TATA-box	48	core promoter element around -30 of transcription start	TATA
ABRE	1	*cis*-acting element involved in the abscisic acid responsiveness	ACGTG
ARE	1	*cis*-acting regulatory element essential for the anaerobic induction	AAACCA
AT1-motif	1	part of a light responsive module	AATTATTTTTATT
GATA-motif	2	part of a light responsive element	AAGATAAGATT
ATCT-motif	1	part of a conserved DNA module involved in light responsiveness	GAATCTAAGCC
Box 4	2	part of a conserved DNA module involved in light responsiveness	ATTAAT
G-Box	1	*cis*-acting regulatory element involved in light responsiveness	CACGTT
ERE	6	ethylene-responsive element	ATTTTAAA
CGTCA-motif	2	*cis*-acting regulatory element involved in the MeJA-responsiveness	CGTCA
TGACG-motif	2	*cis*-acting regulatory element involved in the MeJA-responsiveness	TGACG
O^2-site	1	*cis*-acting regulatory element involved in zein metabolism regulation	GTTGACATGA
